# Long-Read Amplicon Sequencing of Nitric Oxide Dismutase (*nod*) Genes Reveal Diverse Oxygenic Denitrifiers in Agricultural Soils and Lake Sediments

**DOI:** 10.1007/s00248-020-01482-0

**Published:** 2020-01-27

**Authors:** Baoli Zhu, Zhe Wang, Dheeraj Kanaparthi, Susanne Kublik, Tida Ge, Peter Casper, Michael Schloter, Tillmann Lueders

**Affiliations:** 1 Chair of Ecological Microbiology, Bayreuth Center of Ecology and Environmental Research (BayCEER), University of Bayreuth, Dr.-Hans-Frisch-Straße 1-3, 95440 Bayreuth, Germany; 2 Research Unit for Comparative Microbiome Analysis, Helmholtz Zentrum München, Neuherberg, Germany; 3 Key Laboratory of Agro-ecological Processes in Subtropical Region & Changsha Research Station for Agricultural and Environmental Monitoring, Institute of Subtropical Agriculture, Chinese Academy of Sciences, Changsha, Hunan China; 4 Leibniz-Institute of Freshwater Ecology and Inland Fisheries, Stechlin, Germany

**Keywords:** PacBio SMRT sequencing, Oxygenic denitrification, Oxygenic denitrifier, Nitric oxide dismutase (Nod)

## Abstract

Microorganisms play an essential role in nitrogen cycling and greenhouse gas emissions in soils and sediments. The recently discovered oxygenic denitrifiers are proposed to reduce nitrate and nitrite via nitric oxide dismutation directly to N_2_ and O_2_. So far, the ecological role of these microbes is not well understood. The only available tool for a targeted study of oxygenic denitrifiers is their respective maker gene, nitric oxide dismutase (*nod*). Here, we established the use of PacBio long-read sequencing of *nod* gene amplicons to study the diversity and community structure of oxygenic denitrifiers. Two distinct sets of environmental samples, agricultural soil and lake sediment, were investigated as examples. The circular consensus sequences (*ca* 1.0 kb) obtained covered most substitution characteristic of NO dismutase and allowed for reliable classification of oxygenic denitrifiers. Distinct *nod* gene pools and community structure were revealed for the different habitats, with most sequence types affiliated to yet unidentified environmental *nod* lineages. The abundance of *nod* genes ranged 2.2 × 10^6^–3.2 × 10^7^ gene copies g^−1^ soil or sediment, accounting for up to 3% of total bacterial 16S rRNA gene counts. This study indicates that *nod*-gene-targeted long-read sequencing can be a powerful tool for studying the ecology of these novel microbes, and the results also suggest that oxygenic denitrifiers are prevalent and abundant in different terrestrial samples, where they could play an important, but yet overlooked role in nitrogen transformations.

**Electronic supplementary material:**

The online version of this article (10.1007/s00248-020-01482-0) contains supplementary material, which is available to authorized users.

Microbes play important roles in biogeochemical cycling of nitrogen (N), which is often a limiting nutrient for agricultural production. Fertilization shapes soil microbial diversity and community structure, while microbes in turn affect the fate of applied fertilizers [1]. Microorganisms have been recognized as the main drivers for nitrogen loss and soil N_2_O emission, which constitutes the dominant N_2_O source, emitting annually over 4 Tg N_2_O-N into the atmosphere [2]. Various microbial groups involved in N cycling have been studied (e.g. [3]), typically using different activity-based or marker gene-based approaches. Well-established primer systems and PCR assays are available for many marker genes of microbial N cycling, and have been extensively utilized in next-generation sequencing studies (e.g. [4]).

Oxygenic denitrification is a recently proposed N-transforming process, where nitric oxide (NO) is directly disproportionated into N_2_ and O_2_, thus avoiding the powerful greenhouse gas, N_2_O emissions. The reaction is catalyzed by a NO dismutase (Nod) [5]. The use of *nod* gene as a marker for oxygenic denitrifiers was recently established [6, 7], with which, evidence was obtained suggesting that oxygenic denitrifiers could be widespread and phylogenetically diverse [6, 8]. However, the high diversity inferred from *nod* genes provided no clue to the actual identities of these novel oxygenic denitrifiers [6]. So far, the identification of oxygenic denitrifiers and analysis of their community structures solely depend on the length and quality of obtained *nod* sequences. In previous studies, long *nod* sequences were obtained via laborious and low-throughput Sanger sequencing, since next-generation sequencing often generates short reads. Recently, PacBio SMRT sequencing provides access to high-throughput long-read environmental sequence data, but has been applied mostly to metagenomics and full-length 16S rRNA gene analysis (e.g. [9, 10]). Here, we provide a first proof-of-principle for the use of *nod*-targeted PacBio sequencing to study the distribution and community structure of oxygenic denitrifiers in terrestrial systems. Soils, especially under N fertilization, are known as hotspots of microbial N cycling; thus, agricultural soils undergoing different long-term fertilization regimes were chosen as example environments. To demonstrate the wide applicability of this approach, several sediment samples from lakes with different trophic status were also queried (Table 1).

**Table 1:** Soil samples and lake sediments analyzed in this study and their key geochemical characteristics

Sample type	Designation	Explanation	pH	Total nitrogen	SOC/DOC	Trophic status	Reference
Agriculture soil	CF	Soil received chemical fertilizer	5.5	1.9 g/kg	17.5 g/kg		[13]
	CF-straw	Soil received chemical fertilizer and straw	6.0	2.6 g/kg	25.5 g/kg		
	CF-manure	Soil received chemical fertilizer and manure	5.7	2.2 g/kg	22.0 g/kg		
	control	Soil received no extra fertilizers	5.4	1.8 g/kg	18.3 g/kg		
Lake sediment*	Stechlin	Deep, dimictic Lake Stechlin, Germany	7.8	0.2 (mg/l)	4.3 mg/l	meso-oligotrophic	[14]
	Dagow	Lake Dagow, Germany	8.1	0.5 (mg/l)	*n.d*	eutrophic	[11]
	SW	South-west basin of Lake Grosse Fuchskuhle	4.5	1.0 (mg/l)	3205 mg/l	dystrophic	[12]
	NE	North-east basin of Lake Grosse Fuchskuhle	5.8	1.7 (mg/l)	16.4 mg/l	dystrophic	

^*^Data are the average of the bottom water column in March–May 2016

*n.d* no data

Soil from the top layer (0–10 cm) of several agricultural fields with and without long-term fertilizations in Ning-Xiang, China [13], and sediments from a meso-oligotrophic, an eutrophic and a dystrophic lake in Germany [11, 12, 14], were sampled (Table 1). The abundance of *nod* genes and bacterial 16S rRNA was quantified by qPCR, representing respective measure of putative oxygenic denitrifiers and total bacteria in these samples. In all soils, the respective counts of oxygenic denitrifiers (1.12 × 10^7^–1.54 × 10^7^ copy *nod* g^−1^ soil) and total bacteria (from 9.75 × 10^8^ to 1.37 × 10^9^ copies 16S rRNA g^−1^ soil) were similar (Fig. 1b), resulting in a rather comparable relative abundance of *nod* genes at 1–1.3%. *nod* gene abundance was in a similar range as that of *nirK* and *nirS* genes observed in other soil ecosystems [15, 16], indicating that oxygenic denitrifier abundance can be similar to that of conventional denitrifiers. In contrast to conventional nitrifying and denitrifying microbes [16, 17], oxygenic denitrifier abundance examined here seemed not significantly influenced by nitrogen fertilization. While *nod* gene abundances varied more widely in lake sediments, from 2.17 × 10^6^ g^−1^ sediment in Lake Grosse Fuchskuhle to 3.2 × 10^7^ g^−1^ sediment in Lake Stechlin, accounting for about 0.4–3.0% of total bacterial number (Fig. 1b).

**Fig. 1:**
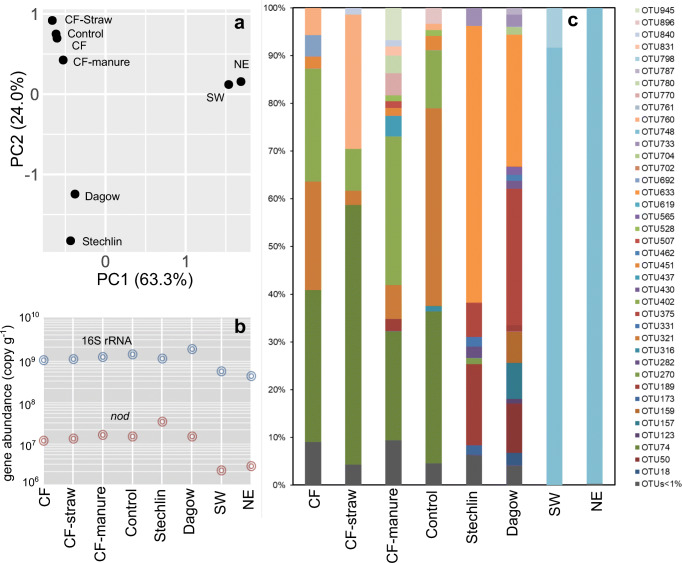
**a** Principal component analysis (PCA) of the oxygenic denitrifier community in all samples, based on the occurrence and relative abundance of all *nod* OTUs. **b** The abundance of total bacteria and oxygenic denitrifiers in different samples as quantified by qPCR targeting 16S rRNA and *nod* genes, respectively. **c***nod* community in different environments. OTU was categorized based on a 90% similarity of *ca*. 1.0 kb *nod* gene fragments. The sample designations are the same as in Table 1

With PacBio SMRT sequencing, long *nod* amplicons (*ca.* 1.0 kb) from all samples were sequenced. Details on the procedure and data analysis are provided in the supplementary information (SI). In total, 61 *nod* OTUs with at least 50 sequences were classified based on 90% similarity on nucleotide level [7]. Representative sequences were used for phylogenetic analysis, revealing that almost all OTUs detected in this study were related to previously reported *nod* lineages [6], with most of them belonging to the *nod* “Aquifer cluster,” 5 OTUs in the “NC10 cluster,” and 1 OTU in the “Reactor cluster 1” (Fig. 2). All representative sequences of these OTUs that related to *nod* clusters possessed previously identified Nod-characteristic residual substitutions (Fig. 3).

**Fig. 2:**
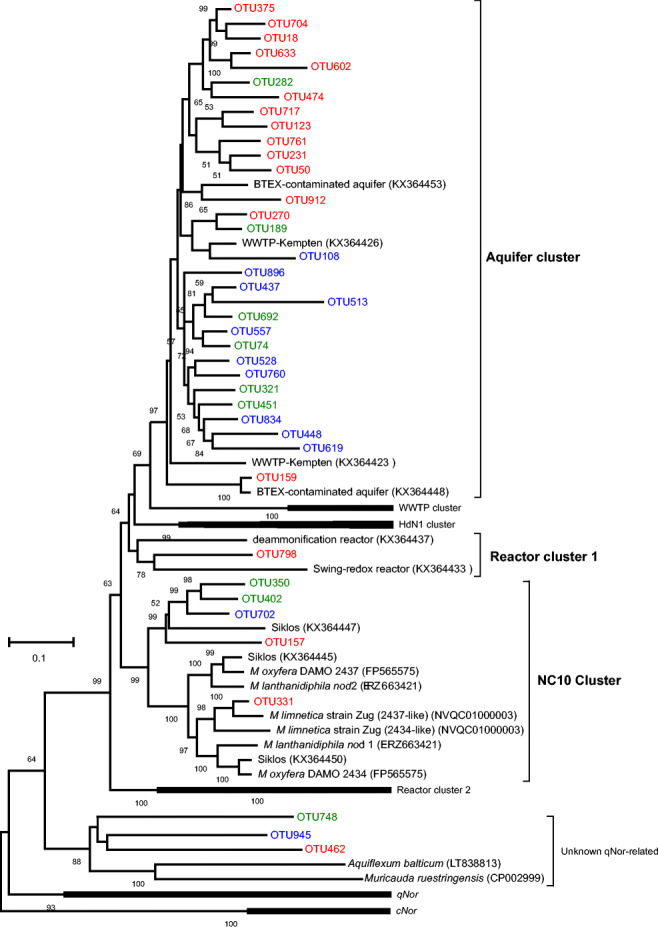
Bootstrapped neighbor-joining phylogeny of *nod* OTU representative nucleotide sequences. Bootstrap support (1000 replicates) greater than 50% is indicated at the nodes. Nod clusters (same as proposed in [6]) containing soil and lake *nod* OTUs are shown in bold. OTUs only detected in soil are shown in blue, lake-specific OTUs in red, and OTUs comprising sequences from both soil and lake sediment are shown in green. The scale bar represents 10% nucleotide sequence divergence. The phylogeny was calculated in MEGA-X

**Fig. 3:**
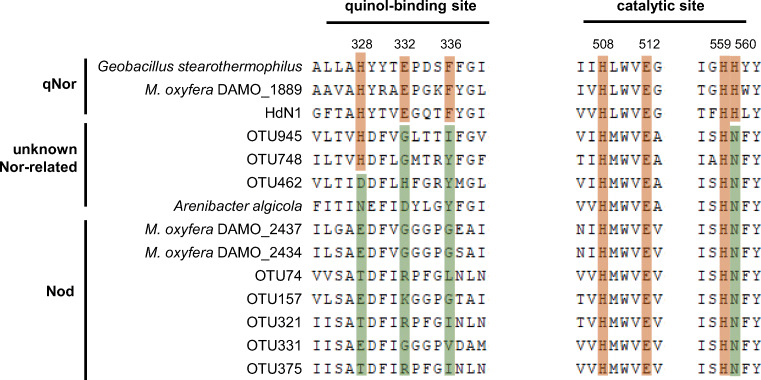
Multiple-sequence alignment of Nod representatives (deduced from *nod* gene sequences) and qNor enzymes around the quinol-binding site and the catalytic site of qNor. The conserved residues for quinol binding and catalytic functioning in qNor are highlighted in orange, whereas substitutions at these sites in Nod and unknown Nor-related sequences are shown in green. Due to the length limit of the recovered Nod sequences, the functional sites shown here are incomplete. Signature residual mutations are observed in soil and lake Nod sequences as well. The residual numbering was according to the *Geobacillus stearothermophilus* qNor (AB450501). The alignment was generated with ClustalW in MEGA-X

Overall *nod* gene pools appeared similar in the agriculture soils subjected to different fertilization regimes (Fig. 1a, c). However, the soil receiving both chemical fertilizer (CF) and manure harbored the highest *nod* diversity across all samples (Fig. 1c). Lake Stechlin and Lake Dagow sediments hosted a higher *nod* diversity compared with Lake Grosse Fuchskuhle (Fig. 1c), which was mostly dominated by OTU748. Possibly, this was related to their different pH and trophic status (Table 1). Principal component analysis (PCA) of *nod* composition clearly supported that distinct oxygenic denitrifier communities were present in agriculture soils and lake sediments (Fig. 1a). Only a limited number of OTUs were shared between soils and sediments, yet habitat-specific OTUs were evident in both soil and lakes, and they tended to be closely placed within the “Aquifer cluster” on the phylogenetic tree (Fig. 2), such as OUT74 was highly abundant in all soils but was not detected in sediments, suggesting that the environment largely determines the composition of *nod* gene pools.

Nevertheless, three OTUs, OTU462, 748, and 945, clustered more closely to the “unknown-qNor-related” sequences [6] in phylogenetic analysis (Fig. 2). Although OTU462 sequences also showed the presumed characteristic residual substitutions for Nod (Fig. 3), the functional connotation of this gene lineage must be interpreted with caution. This suggests that both phylogenetic and residual substitution analysis are necessary for identifying environmental *nod* sequences, a result clearly facilitated by the long sequences obtained from PacBio SMRT sequencing.

Taken all together, the results indicated that agriculture soils harbor diverse and abundant oxygenic denitrifiers, which have been overlooked in the past. Low pH in Lake Grosse Fuchskuhle seemed to disfavor oxygenic denitrifiers. Moreover, functional-gene-targeted long-read sequencing was proven to be a powerful tool for analyzing novel microbial guilds in the environment, and it will allow us to gain better insights into the influencing environmental factors that shape the distribution and community structure of oxygenic denitrifiers and uncover their ecological roles in N cycling in natural habitats.

## Electronic Supplementary Material

ESM 1 (DOCX 287 kb)
