# Supplementary material for: Long-Read Amplicon Sequencing of Nitric Oxide Dismutase (nod) Genes Reveal Diverse Oxygenic Denitrifiers in Agricultural Soils and Lake Sediments
Source: Microb Ecol. 2020 Jan 27;80(1):243–7. doi: 10.1007/s00248-020-01482-0 (PMC7338807; doi:10.1007/s00248-020-01482-0)
Supplement: Supplementary file 1 — (DOCX 287 kb) [file 248_2020_1482_MOESM1_ESM.docx]

**Long-read amplicon sequencing of nitric oxide dismutase (*nod*) genes reveal diverse oxygenic denitrifiers in agricultural soils and lake sediments**

Baoli Zhu^a^ Zhe Wang^a^, Dheeraj Kanaparthi^a^, Susanne Kublik^b^, Tida Ge^c^, Peter Casper^d^, Michael Schloter^b^, Tillmann Lueders^a^

^a^ Chair of Ecological Microbiology, Bayreuth Center of Ecology and Environmental Research (BayCEER), University of Bayreuth, Germany

^b^ Research Unit for Comparative Microbiome Analysis, Helmholtz Zentrum München, Neuherberg, Germany

^c^ Key Laboratory of Agro-ecological Processes in Subtropical Region & Changsha Research Station for Agricultural and Environmental Monitoring, Institute of Subtropical Agriculture, Chinese Academy of Sciences, Hunan, China

^d^ Leibniz-Institute of Freshwater Ecology and Inland Fisheries, Stechlin, Germany

# Supplementary material and methods

### Soil and lake sediment sampling

The Ning-Xiang long-term fertilization experiment station (E 111°54'−112°18', N 28°07'−28°37') locates in the subtropical region of Hunan Province, China. Since 1986 four different fertilization schemes were started: 1, Control, no additional fertilizer; 2, Chemical fertilizer (CF), urea, superphosphate and potassium chloride are applied; 3, CF-straw, chemical fertilizer combined with rice straw; and 4, CF-manure, 70% CF and 30% chicken manure. The cropping system was milk vetch-rice-rice rotation in each year. The total amounts of fertilizer application per hectare in all treatments were 142.5 kg N, 23.2 kg P, 52.3 kg K at early season and 157.5 kg N, 18.6 kg P, 67.2 kg K at the late rice season. Rice straw from the straw plots was returned to the corresponding plots in July and November after the early and late rice harvests at the rates of 2.8 T ha^-1^ and 3.6 T ha^-1^, respectively. Soils were sampled in April 2017 (before the early rice transplanted) from each treatment at four replicate plots. In each 33 m^2^ plot, five soil cores were taken from the upper layer (0−10 cm) and mixed thoroughly.

Lake sediments (*ca*. 0 - 10 cm) were collected in May 2016 using a bottom grab. The sediments were filled up in Schott bottles, sealed and transported to the lab within 24 hours and then stored at 4 ^o^C. Subsamples (*ca*. 1.5 ml) from each sediment were frozen (-20 ^o^C) in duplicate in Eppendorf tubes for future DNA isolation and molecular analysis. The lakes water column geochemistry was bi-weekly monitored, and the average data of the bottom part of their respective water column from March to May 2016 were presented in Table 1.

### DNA isolation

DNA of soils and lake sediments was isolated as previously described (Pilloni, Granitsiotis et al. 2012) with minor modification, the final DNA precipitation was done at 4 ^o^C instead of 20 ^o^C. About 0.4 g sample was used for each DNA extraction, and two extractions were performed from each sample. DNA concentration and quality were checked with the Quant-iT PicoGreen dsDNA Assay Kit (Thermo Fisher, Waltham, USA) on a MX3000p cycler (Agilent, Santa Clara, USA) and by standard agarose gel electrophoresis. Two DNA extracted from the same sample were pooled together for subsequent analysis.

### Pacbio sequencing and microbial community analysis

General *nod* primers nod684Fv2 and nod1706Rv2 (Zhu, Bradford et al. 2017) tailed with Pacbio universal sequence (Table S1) were used in the first round of PCR to amplify *nod* gene fragments from all DNA samples. Each 25 μl reaction contains 1 x KAPA HiFi buffer (KAPA Biosciences, Norway), 0.3 mM dNTPs, 0.3 μM of each primer, 0.75 U KAPA HiFi DNA polymerase (KAPA Biosciences, Norway), and 1 ng template. PCR cycle consists of denaturation at 95 ^o^C for 30 seconds, annealing at 57 ^o^C for 30 seconds and extension at 72 ^o^C for 60 seconds, and in total no more than 23 cycles were performed.

Table S1. *nod* primers used for constructing Pacbio libraries


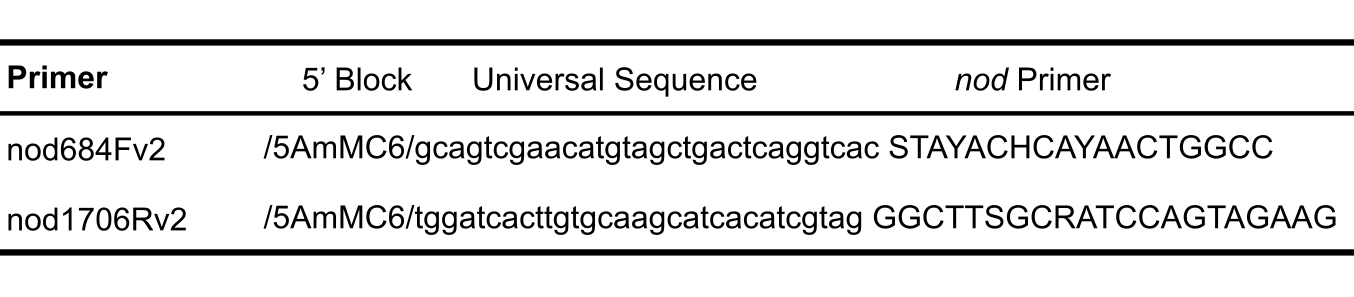


The products of the first round PCR were purified with QIAquick PCR purification kit (QIAGEN, Germany) according to the manufacture’s instruction. The size and concentration of purified PCR products were analyzed on Fragment analyzer (Agilent, US).

For second round PCR, each 25 μl reaction contained 1 x KAPA HiFi buffer (KAPA Biosciences, Norway), 0.3 mM dNTPs, 0.3 μM barcoded universal primer (PacBio barcoded Universal F/R primers plate), 0.5 U KAPA HiFi DNA polymerase (KAPA Biosciences, Norway), and 1 ng first round PCR product as template. PCR cycle consists of denaturation at 95 ^o^C for 30 second, annealing at 57 ^o^C for 30 second and extension at 72 ^o^C for 60 second, and in total no more than 20 cycles were performed. The barcoded amplicons were purified with AMPure PB bead kit (PacBio biosciences, California, US), following the manufacture’s manual. And then their quality and concentrations were checked using an Agilent 2100 Bioanalyzer system (Agilent, US). Since high sequencing depth is unnecessary for profiling the diversity of nod genes, for each SMRT cell 20 barcoded amplicons were pooled together in equal molar quantities, and the total combined mass was about 1000 ng. Repair DNA damage and ends, further purification and SMRT bell library generation were conducted according to PacBio manuals.

### Nod community analysis

After sequencing, the SMRTLink platform was used to generate CCS reads and demultiplex sequences, and convert the .bam files to .fastq files.

Then the sequences in each sample were filtered by their size in Geneious. Sequences with the size out of 1023 ± 50 bp were removed. After that the PacBio universal tail sequences were trimmed from both end of the rest of sequences (min. match length 15 bp, 5 mismatches were allowed). Then the sequences were filtered again by the size window of 1023 ± 20 bp. The remaining sequences from each sample were saved in .fasta files. Then all remaining sequences were pooled together for OTUs picking using QIIME with 90% similarity cutoff. To reduce false diversity introduced by PCR and sequencing errors, OTUs with fewer than 50 sequences were discarded.

In total, 80 OUTs with ≥ 50 sequences were classified. The representative sequences from each remaining OUT were checked by blast in NCBI, and sequences with no significant hit or with low coverage (< 70%) were discarded as well, resulting in 61 OTUs. The relative abundance of each OTU in each sample was calculated and OTU with relative abundance less than 1% was merged together when community plot figure was calculated. While the comparison of nod communities among different samples, all OTUs were taken into account. The sequencing results and number of OTUs in each sample are summarized in Table S2. PCA was calculated in R, using ggbiplot.

Table S2. Summary of sequencing results and number of OTUs in each sample.


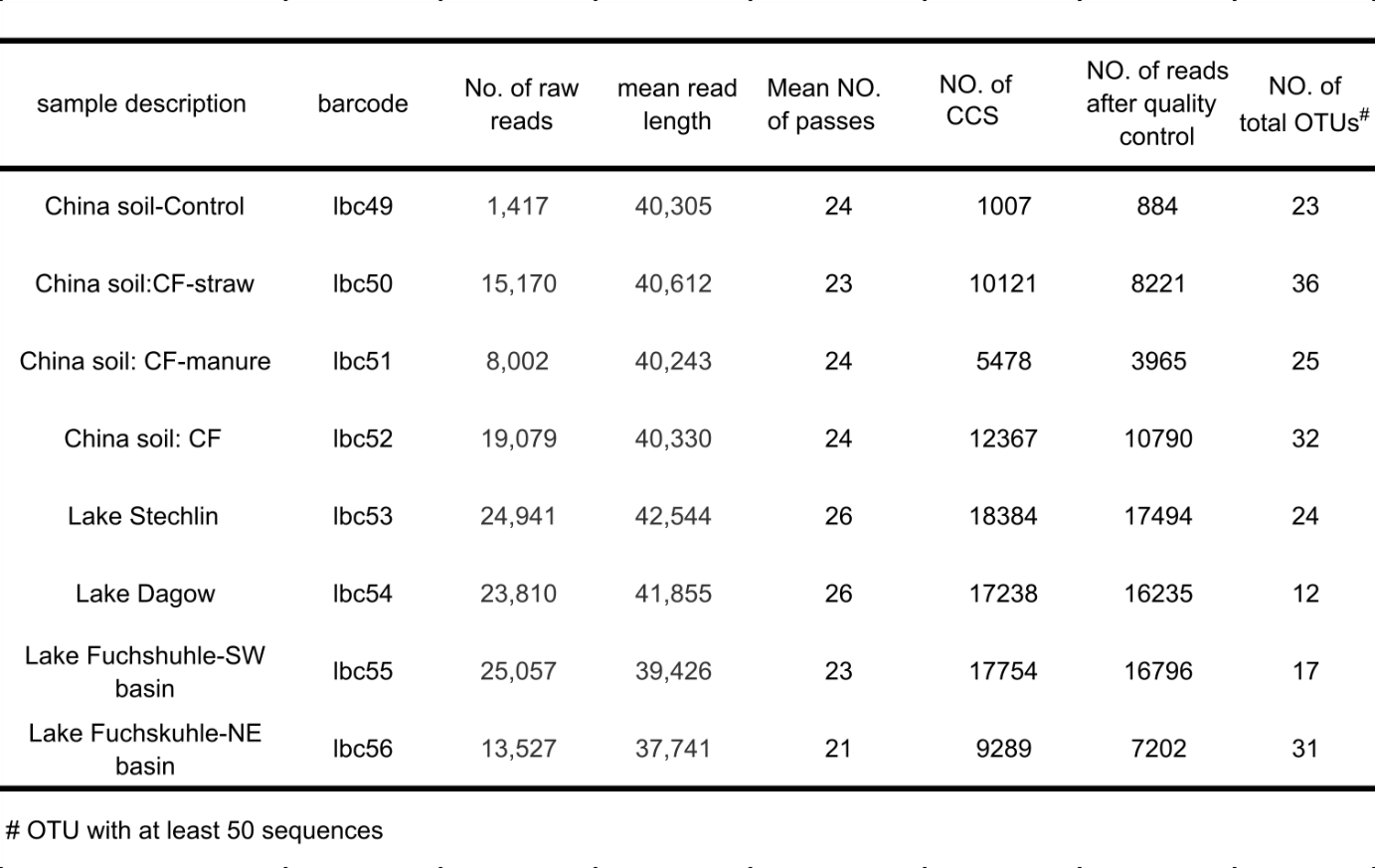


### Quantitative PCR targeting bacterial 16S rRNA and nitric oxide dismutase (*nod*) genes

To quantify the oxygenic denitrifiers and their relative abundance in each sample, qPCR targeting *nod* as well as bacterial 16S rRNA gene was performed, using primer pairs nod1446F & nod1706Rv2 and Ba519F & Ba907R (24), respectively. Synthetic *nod* gene (440 bp, *M. oxyfera* DAMO_2437) and *E. coli* 16S rRNA gene (980 bp) fragments (gBlocks, Integrated DNA Technologies, Leuven, Belgium), covering the respective primer sites with > 60 bp flanking region at each end, were used as respective standards for *nod* and 16S rRNA. Sample DNA in 10- and 100-fold dilutions for *nod*, and in 100- and 1000-fold dilutions for 16S rRNA was quantified. Standard and samples were quantified in triplicates, repeating at least two times. qPCR experiments (25 μl reaction volume) were carried out with MX3000p cycler (Agilent, Santa Clara, USA). 2x Takyon SYBR master mix (Eurogentec, Cologne, Germany) with Rox as the reference dye was used. The annealing temperatures used for *nod* and 16S rRNA qPCR were 57 ^o^C and 52 ^o^C, respectively. qPCR results with PCR efficiencies of 100±10 % were used for calculating gene copy numbers. Absolute *nod* and 16S rRNA gene counts of each sample were calculated per g of soil or sediment.

Representative OTU sequences were translated to amino acids in MEGA-X and then aligned with selected qNor, cNor and previously reported environmental Nod sequences with the ClustralW algorithm with default settings. A phylogenetic tree was constructed based on the amino acid alignment using the neighbor-joining method in MEGA-X. The robustness of the tree topology was tested by bootstrap analysis (1,000 replicates).

### Nucleotide sequence deposition

Sequencing data are deposited with NCBI under the SRA accession number PRJNA596123.
